# Increasing Age at Radical Prostatectomy: A Total Population Analysis in Germany from 2006 to 2022

**DOI:** 10.1245/s10434-025-18740-5

**Published:** 2025-11-19

**Authors:** Martin Baunacke, Johannes Huber, Lennard Haak, Christian Thomas, Christer Groeben

**Affiliations:** 1 Department of Urology, University Hospital Carl Gustav Carus, TU Dresden, Dresden, Germany; 2 Department of Urology, University of Heidelberg, Heidelberg, Germany; 3 Department of Urology, Philipps-University Marburg, Marburg, Hessen, Germany

**Keywords:** Radical prostatectomy, Prostate cancer, Age, Health care, Epidemiology, Population-based study, Germany

## Abstract

**Background:**

With increasing life expectancy, the number of elderly patients seeking curative treatment for prostate cancer is rising. Radical prostatectomy (RP), particularly the robotic approach, has proven feasible in older men. This study analyzes trends in RP among men aged ≥ 75 years of age in Germany between 2006 and 2022.

**Materials and Methods:**

This nationwide retrospective study used German hospital billing data, including 444,102 RPs from 2006 to 2022. Outcomes were in-hospital mortality, transfusion rates, and the length of stay.

**Results:**

The mean age of RP increased from 64.96 ± 6.07 years in 2006 to 66.53 ± 6.88 years in 2022 (*p* < 0.001). The proportion of men of men ≥ 75 years rose from 3% (935 of 28,374) to 12% (3505 of 29,363) (*p* < 0.001). In 2022, patients ≥ 75 had longer hospital stays (8.15 ± 4.63 versus 7.57 ± 3.97 days; *p* < 0.001), higher mortality (0.3% (9 of 3014) versus 0.1% (26 of 26,349); *p* = 0.003), and higher transfusion rate (5% (188 of 3505) versus 2% (641 of 25,858); *p* < 0.001). Patients ≥ 75 years underwent open RP more frequently than robotic RP (14% (1110 of 7831) versus 11% (2227 of 20,205); (*p* < 0.001)), were more frequently treated in nonuniversity hospitals (76% (2643 of 3462) versus 73% (18,534 of 25,344); *p* < 0.001), and less often in high-volume hospitals (> 199 cases/year) in 2022 (11% (1495 of 13,200) versus 13% (1999 of 15,609); (*p* < 0.001)).

**Conclusions:**

The proportion of people ≥ 75 years is continuing to rise, which leads to more patients with poorer functional outcomes. The longer hospital stays, higher transfusion rates, and mortality are nevertheless within acceptable limits.

**Supplementary Information:**

The online version contains supplementary material available at 10.1245/s10434-025-18740-5.

Prostate cancer is the second most frequent cancer in men worldwide.^1^ The incidence of prostate cancer increases with age.^2^ Localized prostate cancer is characterized by the long period of time between diagnosis and the onset of cancer-related symptoms or death. The guidelines therefore recommend curative therapy for patients with a life expectancy of more than 10 years.^3,4^ In Western countries, there is a demographic trend towards a sharp increase in the number of older people. In Germany, for example, it is estimated that over 25% of the population will be over 67 years old by 2070. While it was 78.5 years for men in 2020, it is estimated to rise moderately to 84.6 years in 2070.^5^ This results in the clinical dilemma of balancing life expectancy with surgical risk and functional outcome. While older studies show higher morbidity for elderly patients with radical prostatectomy (RP),^6,7^ new studies with robotic-assisted RP (RARP) show no difference.^8–11^ A recent multicentric study shows a higher risk of complications in elderly people. However, the absolute risk is still low and clinically insignificant.^12^ It therefore seems appropriate to operate on older patients for this type of surgery. However, when considering functional outcomes, the studies show a predominantly poorer outcome in older patients in terms of erection and continence.^9,13–15^ While erectile function may be impaired in old age anyway, incontinence is a much greater burden for patients as it restricts their everyday lives to a greater extent.^16^ In view of these aspects, the question arises as to how relevant the topic is. Studies from Japan and South Korea show an increase in older patients undergoing radical prostatectomy.^17–19^ The latest Japanese study shows a doubling of patients over the age of 70 undergoing robotic-assisted RP (RARP).^18^ Both Asian countries are similar to Western countries in that they have ageing populations and perform a high number of RARPs. However, there is little data available from Western countries. This data usually comprises analyses from individual centers or sub-analyses of comparative studies relating to surgical or oncological aspects.^20,21^ There are no national evaluations focusing on age and RP from Western countries. The aim of this study is to investigate age trends in radical prostatectomy in recent years, focusing on the group of very elderly patients (≥ 75 years).

## Materials and Methods

The primary data source for this study was the national hospital billing database maintained by the German Federal Statistical Office. Detailed descriptions of data extraction procedures and cohort selection have been provided in previous publications.^22^ Diagnoses were recorded using the ICD-10 (International Classification of Diseases, 10th Revision), while medical procedures were coded according to the OPS (“Operationen- und Prozedurenschlüssel”), the German adaptation of the international procedure classification system. This database is compiled from the annual hospital billing records that German hospitals are legally required to submit to the Federal Statistical Office. All German hospitals were included with exception of facilities that exclusively treat psychiatric or forensic patients, or those serving foreign military personnel without providing care to German civilians. Aside from these exceptions, the dataset is considered comprehensive for the intended research purposes.

Primary inclusion criterion was the diagnosis of prostate cancer (ICD-10: C.61) combined with prostatectomy (OPS: 5-604). In combination, OPS codes 5-987 (use of a surgical robot) and 8800c (transfusion of packed red blood cells) were assessed. Others from the database extracted parameters were patient age, mortality during the inpatient stay, and the overall length of hospital stay. However, no data on comorbidity and tumor stage can be obtained from the database.

To assess the development of age and age group related caseload, we defined age groups. The DRG database provides a categorization of patient age by 5 years. Since the age cut-off of 75 years was relevant for our study (see above), we primarily compared patient cohorts above and below this threshold. For some questions, it was necessary to further subdivide the older patients aged 75 years and over into increments of 5.

Annual hospital caseload categories were defined as very low (< 20), low (20–49), medium (50–99), high (100–199), and very high (≥ 200) according to our previous work concerning caseload development of radical prostatectomies in Germany.^23^

Data were fully anonymized and publicly available; thus, ethics committee approval was not required.

### Statistics

Rates, means, and trends were compared using Wald-tests. In this study, rates and shares of absolute values were used. Rates of relative values are distinctly specified. The results section shows averages and standard deviations. The table shows averages, standard deviations, median, and interquartile range (IQR). For trend analysis over time *t*-tests of the linear regression coefficient of the annual caseload development were used. A *p*-value < 0.05 was regarded as significant. For statistical analysis SAS 9.4 (SAS Institute Inc, Cary, NC) was used.

## Results

### Development of Age Groups 75 Years and Older with Radical Prostatectomy

**Fig. 1:**
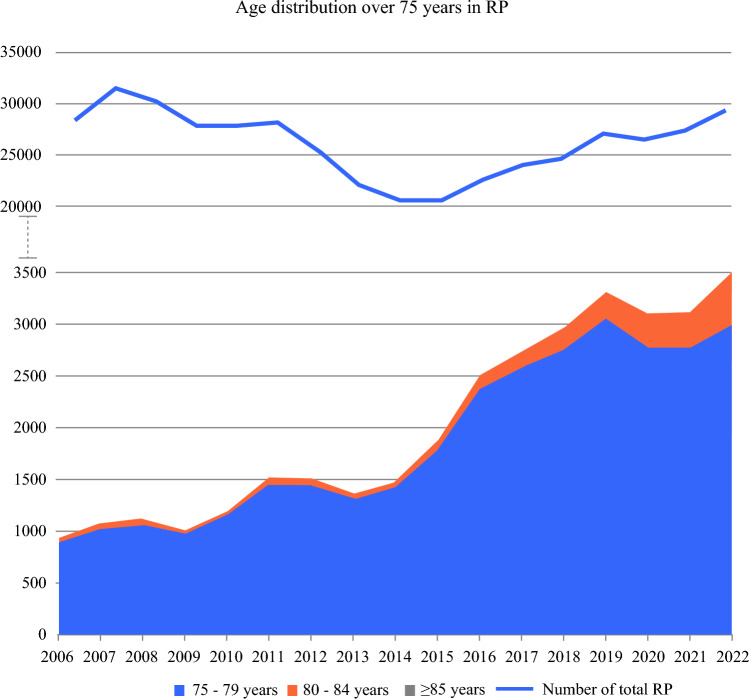
Age distribution of RP patients aged ≥ 75 in Germany from 2006 to 2022 and, in comparison, the total number of RP cases in Germany

We analyzed 444,102 patients who underwent radical prostatectomy in Germany between 2006 and 2022. The average age increased from 64.96 ± 6.07 years in 2006 to 66.53 ± 6.88 years in 2022 (*p* < 0.001). In 2006, the percentage of people aged 75 years and over was 3% (935/28,374). The share quadrupled to 12% (3505/29,363) by 2022 (*p* < 0.001). Compared with the total number of radical prostatectomies, there was a relatively steady increase in patients over 75 years of age. The drop in numbers between 2007 and 2014 is not reflected in patients over 75 years of age (Fig. 1). The proportion of people over 75 years was higher in open radical prostatectomy than in robotic radical prostatectomy in 2022 (14% (1110/7831) versus 11% (2227/20,205); *p* < 0.001). Overall, patients over 75 years received open RP (ORP) more frequently than younger patients (33% (1110/3337) versus 27% (6721/24,699); *p* < 0.001). Patients over 75 years were more frequently treated in nonuniversity clinics (76% versus 73%; *p* < 0.001) (Table 1). The proportion of people over 75 years was lower in high-volume hospitals (> 199 cases/year) than in hospitals with fewer cases (≤ 199 cases/year) in 2022 (11% (1495/13,200) versus 13% (1999/15,609); (*p* < 0.001)).

**Table 1:** Collective of patients with radical prostatectomy over and under 75 years of age in 2022

		All	< 75 years	≥ 75 years	*p*-Value
Radical prostatectomy	Robotic	20,205 (72%)	17,978 (73%)	2227 (67%)	**< 0.001**
	Open	7831 (28%)	6721 (27%)	1110 (33%)	
Population of residence	< 20,000	1715 (6%)	1505 (6%)	210 (6%)	0.4
	20,000–99.999	8457 (29%)	7436 (29%)	1021 (29%)	
	100,000–499,999	10,269 (35%)	9079 (35%)	1190 (35%)	
	≥ 500,000	8364 (28%)	7324 (28%)	1040 (28%)	
	Unknown	558 (2%)	496 (2%)	62 (2%)	
Hospital category (*n* = 28,805)	University	7628 (26%)	6,810 (27%)	818 (24%)	**< 0.001**
	Non-university	21,177 (74%)	18,534 (73%)	2643 (76%)	

### Inpatient Outcome of Age Groups over 75 Years with Radical Prostatectomy

**Fig. 2:**
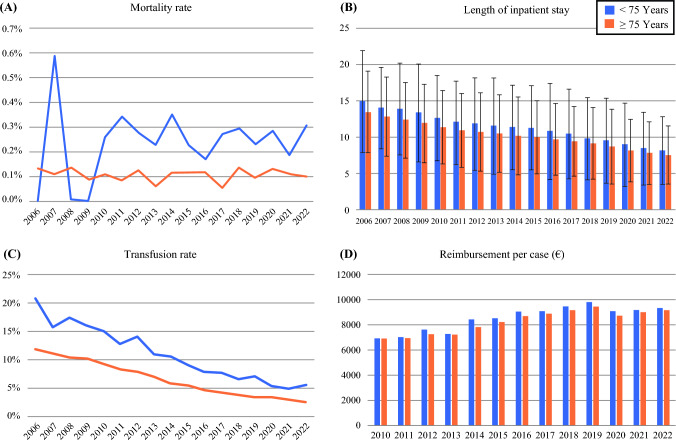
blue: < 75 years and orange: ≥ 75 years; **A** Mortality rate after RP depending on age between 2006 and 2022. **B** Length of inpatient stay in days depending on age between 2006 and 2022. **C** Transfusion rate after RP depending on age between 2006 and 2022. **D** Mean reimbursement in € depending on age with RP between 2006 and 2022

In 2022, the length of inpatient stays for people ≥ 75 was longer than the length of stay for people < 75 years (8.15 ± 4.63 versus 7.57 ± 3.97 days; *p* < 0.001). From 2006 to 2022, the length of stay of people over 75 years of age had decreased to the same extent as the average length of stay (Fig. 2B, Supplementary Table 2). Reimbursement for patients increased from 2010 to 2022 (< 75a: 6900.8 ± 2370 € to 9145.2 ± 1533.3 €; ≥ 75a: 6864.2 ± 898.88 € to 9321.8 ± 2437.8 €) with a significantly higher reimbursement in patients over 75a (9145.2 ± 1533.3 € versus 9321.8 ± 2437.8 €; *p* < 0.001) (Fig. 2D, Supplementary Table 2). The mortality rate for people over 75 was higher than for younger patients (0.3% (9/3014) versus 0.1% (26/26,349); *p* = 0.003). There was no significant change in mortality in both groups from 2006 to 2022 (young: *p* = 0.8, old: *p* = 0.5) (Fig. 2A, Supplementary Table 2). The need for transfusions was significantly higher in patients over 75 years of age than in younger patients (5% (188/3505) versus 2% (641/25,858); *p* < 0.001). From 2006 to 2022, there was a significant reduction in transfusions in both age groups (< 75 a: 21% (193/935) to 5% (188/3505), *p* < 0.001; ≥ 75a: 12% (3224/27,439) to 2% (641/25,858), *p* < 0.001) (Fig. 2C, Supplementary Table 2).

### Subgroups of Patients over 75 Years

Owing to data anonymization regulations, there is very little information on people over 85 years of age. A significant increase in RP was observed in the subgroup of people over 80 years of age. The proportion of 80+ increased tenfold between 2006 and 2022 (0.13% (36/28,374) to 1.67% (491/29,363); *p* < 0.001), whereas the proportion of patients 75–79 years had more than tripled (3.2% (899/28,374) to 10.3% (3014/29,363). Regarding the outcome, it can be seen that the length of stay has improved significantly in both subgroups. While it still differed in 2006 (14.9 ± 7.1 d (75–79a) versus 16.1 ± 6.4 d (80–84a)), it is now almost the same (8.14 ± 4.72 d (75–79a) versus 8.18±4.04 d (80 – 84a). The transfusion rate improved similarly in both groups (75–79a: 20.6% to 5.3% & 80–84a: 22.2% to 5.5%) (Supplementary Table 1).

## Discussion

We analyzed the development of age and the associated perioperative outcome for all cases of radical prostatectomy in Germany over a time span of 17 years. The share of patients age 75 years and higher increased continuously by almost fourfold during that time while the share of patients over 80 years increased by a factor of 10 to almost 2% in 2022. Older patients show elevated perioperative mortality rates, a longer length of stay (LOS), and an increased need for blood transfusion. Radical prostatectomy in patients ≥ 75 years of age increases the overall costs for the hospital stay by a mean of approximately €200 per case. Nevertheless, the differences in mortality and reimbursement are statistically significant but clinically negligible.

Our results showed a decrease in national case numbers for radical prostatectomy from 2006 to 2014 by roughly one third with a continuous incline thereafter until 2022. In the past, this was related to the implementation of active surveillance for low risk tumors at the beginning of the 21^st^ century, with changes in the according national guidelines and a consequential decline of active treatment options in the following years.^22^ In addition, several focal therapy options (e.g. cryotherapy, highly focused intensive ultrasound, and Tookad) could have also taken their share in caseload during that time as shown in previous studies.^24,25^ Interestingly here is the recurrence of active treatment in the past 10 years. This could be explained by economic effects, such as the Gartner-hype-cycle.^25^ demonstrating a reduction of market share when the first “hype” of the new technology or technique has faded, with a return to already established options a few years after market initiation.

Despite the development of overall case numbers for radical prostatectomy, shares for old patients undergoing surgery have continuously increased by threefold for patients > 75 years and over and tenfold for patients > 80 years. It appears that adherence to guidelines regarding life expectancy and treatment planning for prostate cancer is increasingly viewed with flexibility.^3,4,26^ Here, a life expectancy of at least 10 years is required as a prerequisite for the offer of surgical treatment for prostate cancer. Obviously, this threshold is not always respected in everyday clinical practice by patients and their treating urologists. For many elderly patients, life expectancy exceeds 10 years, allowing them to be considered for radical prostatectomy (RP) on the basis of health status rather than biological age, as demonstrated in selected cohorts.^27,28^ Despite an increased risk of perioperative complications and mortality for patients aged ≥ 75 years, well-selected individuals still show favorable outcomes with RP compared with noncurative treatments, which could result in worse cancer-specific mortality.^29^ Health assessment tools, such as the Geriatric 8 (G-8) score, the MiniCog, and the Identification of Seniors at Risk (ISAR) score, help identify fit individuals who may benefit from RP, even in the presence of comorbidities or higher tumor risk, with such tools increasingly applied in German hospitals upon admission.^30^ These findings and general developments could increasingly lead surgeons to offer prostatectomy to patients of advanced age. It must be kept in mind that the average > 80-year-old patient today is not necessarily the same age as a patient around 20 years ago in terms of physical fitness, performance, and previous diseases or surgery. According to numerous studies, the general life expectancy of men in Western industrialized nations increases continuously over time as well as their mean health status.^31,32^ Owing to continuous improvement in living conditions, we frequently see patients of 75 or even 80 years without concerning cardiovascular or respiratory conditions.^31^ In addition, patients are increasingly demanding more participation in the choice of diagnostics and treatment for their diseases.^33,34^ Ultimately, the current EAU-guidelines also recommends to take comorbidity and frailty into account when discussing eligible treatment options for patients with prostate cancer in addition to the chronological age.^3,26^ In times of increasing life expectancy and inclining numbers of older patients demanding active treatment of their disease, this appears to be an ever-reasonable principle for treatment decision-making.

With increasing age risk for complications or even mortality during and after major abdominal surgery inclines continuously. This has been numerously proven in past studies.^23,35–37^ On multivariable analysis, senile age has always shown to be an independent risk factor for perioperative mortality for radical prostatectomy^23^ as well as for other urooncologic surgery. This can be explained by a reduced or even frail physical status and increased risk of perioperative cardiovascular complications. ^38,39^ In our results, we were able to show that mortality rates for patients >75 years had increased threefold compared with younger patients, although mortality rates were overall low (0.1–0.3%) compared with other major abdominal surgery such as radical cystectomy.^35,40^ If this rather marginal difference is clinically relevant, it must certainly be critically discussed. Likewise, we saw approximately twofold higher perioperative transfusion rates which decreased considerably during the investigated timespan from over 10% to less than 5% for all patients. When analyzing the length of hospital stay, we detected an overall decrease for all patients by almost 1 week in 17 years, which is in line with previous investigations on German total population data.^23,35^ Older patients showed longer LOS although the difference decreased over time from 1.5 days in 2006 to almost 0.5 days in 2022. Given this difference in LOS differences in reimbursement for radical prostatectomy and the according hospital stay were seen consistently over time amounting to a mean of €200 more for patients > 75 years. Thus, it can be concluded that there are statistically significant differences between younger and older patients concerning the perioperative outcome and costs, although clinical relevance must be questioned. The share of older patients receiving ORP instead of RARP was slightly elevated compared with younger patients (33% versus 27%, *p* < 0.001). When comparing the respective outcomes, we saw no differences concerning the surgical approach. This is supported by numerous studies that show no relevant differences in the functional outcome of both surgical procedures. There are only a few studies that have specifically evaluated the outcome of elderly patients depending on the surgical procedure.^41^ Nevertheless, earlier studies showed an advantage for the robotic procedure, and the number of studies with similar results is increasing.^42–45^ Basiri et al. discuss, among other things, the fact that increasingly older patients are undergoing robotic surgery, which compensates for the functional outcome through age adjustment.^43^ Thus, it can be concluded, that patients can be offered RARP independent of their age.

The significant increase in older patients undergoing radical prostatectomy must be also discussed in the context of functional outcomes. The rate of erectile function is significantly worse in elderly patients, although there are different studies that found one or no difference between age groups.^13,14^ In view of the increasing rate of erectile dysfunction owing to age in general, the question of relevance arises here.^46,47^ Urinary incontinence should be given greater importance here, as it significantly impairs the everyday lives of those affected.^48^ It has a particularly negative impact on older people affected.^49,50^

Continence recovery rates range from 59–86% at 12 months in ≥ 70 years, versus 69–93% in younger men.^10,13,15,51^ Erectile function rates show a mixed picture, with a tendency towards poorer outcomes in elderly patients 20–44% at 12 months in ≥ 70 years, versus 27–50% in younger men.^10,13,15,51^ Even though the studies cannot be directly compared owing to different definitions of continence, erectile function and collectives, the picture of significantly lower continence rates or erectile function in elderly patients is still evident. In the context of inadequate care for men with urinary incontinence following radical prostatectomy, the question arises as to whether this growing group of elderly patients with a higher risk of incontinence is subsequently receiving adequate treatment.^52^ There is a need for studies to examine whether the increasing number of elderly patients undergoing RP also leads to a deterioration in the overall functional outcome. Alternatively, the question arises as to whether those elderly patients who undergo surgery today are in such good physical condition that they do not experience a poorer functional outcome.

Furthermore, surgical planning and resource allocation must also be discussed in light of this development, given the growing number of elderly patients. Firstly, it should be noted that despite the overall low mortality and transfusion rates, these are still higher than in young patients. This reflects the increase in multimorbidity in old age and should serve as an indication that such patients should be treated in high-volume clinics with sufficient experience.^23^ Secondly, this development must also be viewed critically in light of demographic trends in Western countries. On the one hand, the ageing population is leading to an increase in the number of these patients and thus in the number of radical prostatectomies overall. On the other hand, demographic trends are leading to a decline in the number of medical personnel.^53^ In this respect, we must critically ask whether increasingly competitive medical resources are being claimed by patients who may not benefit from the surgery in terms of their life expectancy.

Our study is the first to analyze age distribution of radical prostatectomy for prostate cancer focusing on the increase of older patients in Germany using total population data. The main study limitations concern the nature of the data itself. Although billing data are highly accurate, tumor characteristics and further clinical details such as comorbidities and ASA data are not included, which limits adjustment for confounding. There is also no information on oncological end points such as margin status and recurrence. Owing to data protection regulations in Germany, single patients or institutions may not be identified from the DRG-database. Therefore, revision or verification of each data set is not possible. Since datasets are only based on one inpatient visit, the further course of events, such as oncological outcome or late complications, could not be assessed.

Given the extensive caseload numbers for this procedure and the possibility to present complete national data, some slight variances and small irregularities appear to be negligible. However, the principal risk of systematic bias has to be kept in mind when interpreting the results.

In conclusion, we can say that adherence to guidelines regarding life expectancy and treatment planning for prostate cancer is increasingly viewed with flexibility. Urologists as well as the treating hospitals will increasingly have to adapt to older patients demanding radical prostatectomy. Presumably, decisive factors for treatment choice will rather be clinical condition (biological age) and comorbidities, instead of chronological age. However, owing to slightly worsened outcome surgery should still be discussed critically with patients > 75 years. Concerning the surgical approach, age does not seem to be an important factor as well. However, the focus should be on critically discussing the functional outcome according to RP in older patients compared with younger patients.

## Supplementary Information

Below is the link to the electronic supplementary material.

Supplementary file1 (DOCX 18 KB)
