# Supplementary material for: Increasing Age at Radical Prostatectomy: A Total Population Analysis in Germany from 2006 to 2022
Source: Ann Surg Oncol. 2025 Nov 19;33(3):2783–90. doi: 10.1245/s10434-025-18740-5 (PMC12901175; doi:10.1245/s10434-025-18740-5)
Supplement: Supplementary file 1 — Supplementary file1 (DOCX 18 KB) [file 10434_2025_18740_MOESM1_ESM.docx]

|  | | **< 75 years** | **75 – 79 years** | **80 – 84 years** | **≥ 85 years** |
| --- | --- | --- | --- | --- | --- |
| ***Distribution of the number of RPs*** | | | | | |
| **Number of RP** | **2006** | 27,439/28,374 (96.7%) | 899/28,374 (3.2%) | 36/28,374 (0.1%) | ***** |
|  | **2022** | 25,858/29,363 (88.0%) | 3,014/29,363 (10.3%) | 491/29,363 (1.7%) | 16/29,363 (0.05%) |
| **Number of robotic RP** | **2006** | 179/182  (98.4%) | 3/182  (1.6%) | ***** | ***** |
|  | **2022** | 17,978/20,205 (89.0%) | 1,920/20,205 (9.5%) | 297/20,205 (1.5%) | 10/20,205 (0.05%) |
| ***case number of the clinic*** | | | | | |
| **>199 RP/year** | **2007*** | 8,346/8,563 (97.5%) | 209/8,563  (2.4%) | 8/8,563  (0.1%) | ***** |
|  | **2022** | 11,705/13,200  (88.7%) | 1,273/13,200 (9.6%) | 222/13,200 (1.7%) | ***** |
| **100-199 RP/year** | **2006** | 7,563/7,877  (96.0%) | 302/7,877  (3.8%) | 12/7,877 (0.2%) | ***** |
|  | **2022** | 4,852/5,585  (86.9%) | 621/5,585 (11.1%) | 112 /5,585  (2.0%) | ***** |
| **50-99**  **RP/year** | **2006** | 7,552/7,818  (96.6%) | 254/7,818  (3.2%) | 12/7,818  (0.2%) | ***** |
|  | **2022** | 4,773/5,471  (87.25%) | 605/5,471  (11.1%) | 90/5,471  (1.6%) | 3/5,471  (0.05%) |
| **<50 RP/year** | **2006** | 4,327/4,502  (96.1%) | 168/4,502  (3.7%) | 7/4,502  (0.2%) | ***** |
|  | **2022** | 3,985/4,553  (87.5%) | 504/4,553 (11.1%) | 64/4,553  (1.4%) | ***** |
| ***Outcome*** | | | | | |
| **Inpatient stay (days)**  mean ± SD  median (IQR) | **2006** | 13.5±5.59 | 14.9±7.1  13 (11 - 17) | 16.1±6.4  16 (11 - 21) | ***** |
|  | **2022** | 7.57±3.97 | 8.14±4.72  7 (6 - 9) | 8.18±4.04  7 (6 - 9) | ***** |
| **mortality** | **2006** | 36/27,475  (0.13%) | ***** | ***** | ***** |
|  | **2022** | 26/26,349  (0.1%) | 9/3014  (0.3%) | ***** | ***** |
| **Transfusion rate** | **2006** | 3,224/27,439 (11.7%) | 185/899  (20.6%) | 8/36  (22.2%) | ***** |
|  | **2022** | 641/25,858  (2.5%) | 161/3,014  (5.3%) | 27/491  (5.5%) | ***** |

**Supplementary Table 1:** Distribution of the collective according to age group. *****Data were not available due to data blinding.

| **Year** | **Total RP** | **Number of RP** | | | | **Mortality rate** | | | | **Transfusion rate** | | | | **Length of inpatient stay in days** | |
| --- | --- | --- | --- | --- | --- | --- | --- | --- | --- | --- | --- | --- | --- | --- | --- |
|  |  | **< 75 years** | | **≥ 75 years** | | **< 75 years** | | **≥ 75 years** | | **< 75 years** | | **≥ 75 years** | | **< 75 years** | **≥ 75 years** |
| 2006 | 28374 | 27439 | 97% | 935 | 3% | 36 | 0,0% | 0 | 0,13% | 193 | 21% | 3224 | 11,7% | 13,5±5,59 | 14,95±7,02 |
| 2007 | 31600 | 30522 | 97% | 1078 | 3% | 33 | 0,6% | 6 | 0,11% | 169 | 16% | 3336 | 11% | 12,85±5,43 | 14,04±5,63 |
| 2008 | 30195 | 29066 | 96% | 1129 | 4% | 39 | 0,0% | 0 | 0,13% | 197 | 17% | 3011 | 10% | 12,36±5,23 | 13,86±6,38 |
| 2009 | 27863 | 26849 | 96% | 1014 | 4% | 23 | 0,0% | 0 | 0,09% | 162 | 16% | 2714 | 10% | 11,91±5,4 | 13,33±6,78 |
| 2010 | 27894 | 26702 | 96% | 1192 | 4% | 29 | 0,3% | 3 | 0,11% | 180 | 15% | 2485 | 9% | 11,41±5,05 | 12,64±5,82 |
| 2011 | 28201 | 26669 | 95% | 1532 | 5% | 22 | 0,3% | 5 | 0,08% | 195 | 13% | 2203 | 8% | 10,95±5,07 | 12,05±5,71 |
| 2012 | 25357 | 23838 | 94% | 1519 | 6% | 29 | 0,3% | 4 | 0,12% | 214 | 14% | 1863 | 8% | 10,72±5,34 | 11,87±6,33 |
| 2013 | 21847 | 20481 | 94% | 1366 | 6% | 12 | 0,2% | 3 | 0,06% | 150 | 11% | 1427 | 7% | 10,53±5,3 | 11,56±6,6 |
| 2014 | 20423 | 18936 | 93% | 1487 | 7% | 22 | 0,3% | 5 | 0,12% | 156 | 10% | 1076 | 6% | 10,19±5,35 | 11,37±5,81 |
| 2015 | 20585 | 18694 | 91% | 1891 | 9% | 22 | 0,2% | 4 | 0,12% | 170 | 9% | 993 | 5% | 10,02±5,03 | 11,28±5,76 |
| 2016 | 22523 | 19999 | 89% | 2524 | 11% | 23 | 0,2% | 4 | 0,11% | 195 | 8% | 925 | 5% | 9,73±4,95 | 10,82±6,63 |
| 2017 | 24001 | 21246 | 89% | 2755 | 11% | 11 | 0,3% | 7 | 0,05% | 211 | 8% | 891 | 4% | 9,44±4,8 | 10,44±6,19 |
| 2018 | 24715 | 21730 | 88% | 2985 | 12% | 30 | 0,3% | 8 | 0,14% | 192 | 6% | 817 | 4% | 9,18±4,94 | 9,8±5,66 |
| 2019 | 27178 | 23849 | 88% | 3329 | 12% | 23 | 0,2% | 7 | 0,10% | 232 | 7% | 801 | 3% | 8,73±5,13 | 9,55±5,83 |
| 2020 | 26551 | 23429 | 88% | 3122 | 12% | 31 | 0,3% | 8 | 0,13% | 163 | 5% | 751 | 3% | 8,17±4,28 | 8,94±5,7 |
| 2021 | 27432 | 24296 | 89% | 3136 | 11% | 27 | 0,2% | 5 | 0,11% | 153 | 5% | 684 | 3% | 7,84±4,33 | 8,47±4,98 |
| 2022 | 29363 | 25842 | 88% | 3521 | 12% | 26 | 0,3% | 9 | 0,10% | 188 | 5% | 641 | 2,5% | 7,57±3,97 | 8,15±4,63 |

**Supplementary Table 2**: Distribution of the collective regarding number of RP, Mortality rate, transfusion rate and length of inpatient stay.
